# Early graft failure following lower extremity bypass

**DOI:** 10.1016/j.jvscit.2024.101642

**Published:** 2024-09-19

**Authors:** Donald T. Baril

**Affiliations:** Division of Vascular Surgery, Cedars-Sinai Medical Center, Los Angeles, CA

The vast majority of this journal's readers have inevitably received a phone call from the emergency department, a fellow or resident, a primary care physician, or even a fellow vascular surgeon that starts with the person on the other side of the phone asking, “Do you remember patient *X*?” Perhaps regrettably, of course, we remember patient *X*, who just a few short weeks ago we spent hours splicing together pieces of marginal vein together to anastomose decent inflow to mediocre outflow for their redo-redo bypass and were frankly a bit surprised that they were discharged with a palpable pulse after making up some special antithrombotic regimen . . only now to be updated that the pulse is no longer there and the foot looks less than amazing. Unfortunately, despite our best evidence demonstrating that patients with chronic limb-threatening ischemia with adequate vein do well compared with their matched endovascular equivalents,^1^ early graft failure after bypass remains an ongoing concern and, when it does occur, it leaves surgeons facing challenging decisions.

In this journal and its partners, there are innumerable amazing descriptors of total endovascular arch repairs, radiation-free four-vessel fenestrated cases, and four-access recanalization of all three tibial vessels with simultaneous endovascular repair of an occluded 10-cm popliteal aneurysm, but it is safe to say that lower extremity bypass remains one of the most challenging technical operations that we perform. Taken in their individual parts, perhaps no one step is all that particularly difficult when compared with some of the other technical maneuvers we might perform, but when you put them all together, you come to realize that all of these steps—including vein harvesting and preparation, tunneling, anastomosing, and wound closure—all need to be essentially perfect to have a bypass work well immediately and over time. Perhaps even more defeating is the fact that, occasionally, you might be perfect (or at least close) and still end up with an early graft thrombosis. That being said, the old adage once told us that graft failure in the first 2 months is most likely technical; however, other factors have been implicated more recently, including vein quality, active smoking, female gender, and more distal targets.^2^^,^^3^ These more recent reviews have demonstrated that “technical” is not limited merely to technique itself, but includes many other factors beyond just doing a good job sewing A to B, not the least of which is surgical judgment.

Like every other vascular surgical procedure we perform, much of the success of lower extremity bypass lies in the planning. When it comes to bypass and trying to optimize outcomes, we know that the major contributor is most often the conduit. If all of our patients were gifted with the perfect single segment great saphenous vein, much of the decision-making initially and, perhaps, after early failure would be easier. However, so many of the conduits we use are less than ideal and might be alternative autologous veins (often spliced together), cryoveins, prosthetic, composites, and so forth.^4^^,^^5^ When these conduits fail early on, surgeons are faced with the dilemma as to whether or not these are then worth salvaging. There are many times we have done the best we can, but even if the conduit was marginal at best, it is worth investigating whether there might be a correctable issue with it. Specifically, is there an issue at either anastomosis (or at a splice point if used), is there a retained valve (or a valve causing an issue even if vein was used in reversed fashion), was the tunnel in fact not quite in the plane that we were aiming for or had more of an angle than desired at one end or the other? Granted, there are other times when, indeed, all of these factors were okay, perchance even a 1-month postoperative duplex ultrasound examination validated that, but the bypass still failed.

Once failure has occurred, the first step is determining, as noted, whether, indeed, that was the best you could do or if, rather, there is something that can be improved upon. Remembering the other old adage—inflow-conduit-outflow—one needs to ascertain whether any one of these are then worth improving upon. Furthermore, it is always important to remember to do no harm; is there another option—endovascular or open—to salvage the limb that might have a higher success rate (and lower risk profile) than trying to reopen the failed bypass?

In prior times, when addressing an early bypass graft failure, we were challenged with open thrombectomies, including how best to pass embolectomy balloons through valves or across anastomoses, but presently, we have a host of endovascular thrombectomy devices, with new iterations of aspiration, mechanical removal, and thrombolytic administration being released seemingly every month. These devices have allowed surgeons to tackle most of these early graft failures with purely percutaneous techniques. Once a flow channel has been reestablished, the culprit lesion is usually uncovered. After this has been identified, attention can then turn to optimizing the inflow or outflow with various endovascular techniques, or treating some portion of the conduit or an anastomosis if needed. Even if endovascular techniques cannot be used to fix the problem safely, visualizing the issue with an angiogram in a patent bypass will generally allow for a focal repair, and thereby minimize the morbidity of unnecessarily reopening surgical wounds beyond what is needed. There are clearly many steps in trying to salvage a failed bypass, like the index operation, which require judgment (and experience to hone that judgment), not the least of which is whether the outflow can be improved upon. Beyond the conduit, particularly in the endovascular era and a time when diabetes is rampant, the outflow quality is often borderline for patients undergoing bypass. Although there may be means to improve upon this with angioplasty and other endovascular techniques or extending a distal anastomosis with patch angioplasty, there are other times when we get what we get and need to determine if it is best not to try and do more.

After an early graft failure has been salvaged, just like the procedure itself, postoperative management must be considered to determine whether something additional can be done to assist with maintaining patency. Antithrombotic regimens after lower extremity bypass are varied at best, and in individual practice are based on a great deal of bias and gut feeling.^6^^,^^7^ That being said, most commonly, if safe for the patient, a more aggressive approach to the antithrombotic regimen after a bypass salvage seems warranted at least in the short term to give the bypass the greatest chance for long-term patency. Additionally, ongoing strict attention to risk factor modification, specifically smoking cessation and glucose control in the perioperative period, may also help in doing everything possible to optimize the future of a failed bypass. Finally, once a bypass has been re-opened, it is imperative to perform duplex ultrasound surveillance at short intervals (consider 1-month postoperatively and subsequent 3-month intervals thereafter for ≤1 year) and to intervene as necessary if novel stenoses arise or if a previously noted issue recurs.^8^

## Conclusions

Despite all of the advances in lower extremity endovascular therapies over the past two decades, it is evident that surgical bypass is here to stay and will always have a role in limb salvage. Unfortunately, along with this, we will always need to be prepared to treat the occasional early graft failure. Luckily, with the increasing armamentarium of endovascular tools, many of these can be managed with minimally invasive techniques and furthermore, improved longer term outcomes might be achievable with ongoing comprehensive vascular care.

## Disclosures

None.
